# A Rare Case of Groove Pancreatitis

**DOI:** 10.7759/cureus.21109

**Published:** 2022-01-11

**Authors:** Benjamin Sinyor, Bethsy Daverman, Laura Sanchez, Pulkit Arora

**Affiliations:** 1 Family Medicine, North Florida Regional Medical Center, Gainesville, USA

**Keywords:** groove pancreatitis, pancreatitis causes, general radiology, anatomy & physiology, rare entity

## Abstract

One of the most commonly diagnosed gastrointestinal disorders in the inpatient hospitalization setting is pancreatitis. It is increasingly common in the western world and the incidence is only growing over the last decade. The most common causes of pancreatitis are gallstones, alcohol, and hypertriglyceridemia. However, we present a very unusual case of pancreatitis caused by a unique anatomical obstruction called groove pancreatitis (GP). It is extremely infrequent as there is no prevalence, and can often be mistaken for pancreatic cancer. We explore the case of a 36-year-old male with no significant risk factors of pancreatitis presenting with abdominal pain and was found to have groove pancreatitis.

## Introduction

Groove pancreatitis (GP) is an atypical form of chronic pancreatitis. Its name is derived from the area which it affects i.e., the “groove” between the duodenum, head of the pancreas, and the common bile duct. Patients commonly present with symptoms of duodenal obstruction, including vomiting, and rarely jaundice [1]. Men in their 40s and 50s with a history of chronic alcoholism are typically affected [2]. A handful of cases suggest that pancreatitis in the groove area may arise from obstruction in the ductal system, eventually leading to fibrosis, stasis, and ultimately inflammation of surrounding structures [1]. Two forms of GP have been described, although a discernable difference may not be readily apparent. The pure form is characterized by scar tissue affecting the pancreatic groove. Alternatively, the segmental form involves the head of the pancreas with stenosis at the major pancreatic duct as well as scarring of the groove [2]. Scarring in this area may present with features that mimic pancreatic adenocarcinoma. Scar tissue may lead to compression of blood supply, stenosis in the bile duct, and rigidity in the duodenal wall [1]. Of notable importance is that while GP can mimic, it may also coexist and/or mask adenocarcinoma [2]. Distinction on imaging may often be difficult. The treatment of choice is conservative management, although symptom severity coupled with the need to rule out malignancy, often leads to surgical management [3]. Since there is a low incidence of groove pancreatitis partially due to lack of familiarity with the disease, our findings can hopefully elucidate another cause of pancreatitis and inform physicians of an extraordinary case that may help to differentiate from pancreatic cancer or other etiologies.

## Case presentation

A 36-year-old male with a past medical history of posttraumatic stress disorder (PTSD), presented to the emergency department with abdominal pain for eight hours. He woke up with non-radiating, constant, throbbing-type epigastric pain associated with nausea. He denied any aggravating or relieving factors. Of note, he had no surgical history and his family is devoid of pancreatitis, pancreatic cancer, irritable bowel syndrome, or gastrointestinal diseases. He also reported no extensive alcohol use and that he drinks less than one drink a week. He denied any recreational use and has never smoked. As the day progressed, the pain intensified which he then notified emergency medical services. He received fentanyl 100 mcg which eased the pain from 10/10 to 6/10. He denied the use of non-steroidal anti-inflammatories (NSAIDs), BC or Goody powder. His home medications were buspirone 15 mg daily for anxiety, clonazepam 0.5 mg as needed for sleep, and intramuscular testosterone injection every two weeks for the past two months. Per our gastroenterologist, none of the above lists of medications have been shown to cause pancreatitis. 

**Figure 1:**
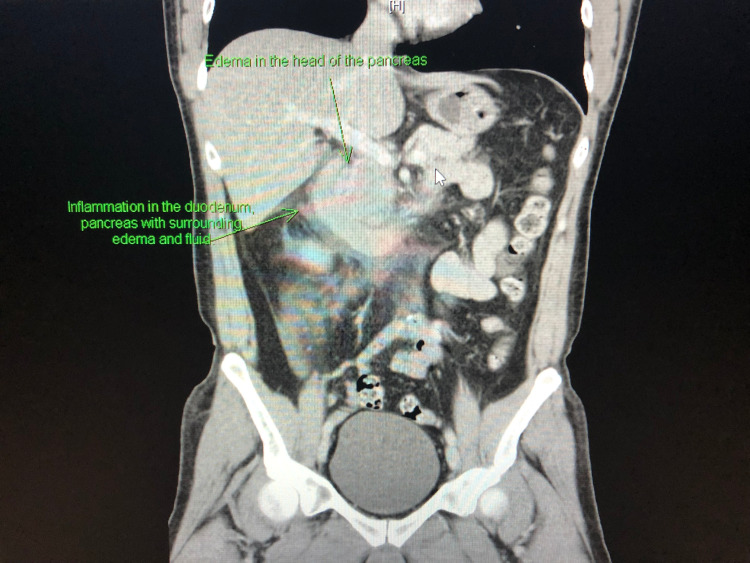
CT of the abdomen in a coronal view Computed tomography (CT) of the abdomen displaying edema at the head of the pancreas. There is also inflammation in the duodenum and pancreas with surrounding fluid and edema.

Upon arrival at our facility, his vitals were within normal range. Physical exams were unremarkable except for bilateral upper quadrant abdominal tenderness, and hypoactive bowel sounds. Relevant laboratory findings were as follows: creatinine 1.10 mg/dL, aspartate transaminase 52 units/L, alanine transaminase 146 units/L, lipase 1723 units/L, triglycerides 249 mg/dL, lactate dehydrogenase 189 units/L, complete blood count, and immunoglobulin (IgG4) were within normal range. The first image being done was an ultrasound of the abdomen. The abdominal ultrasound demonstrated mild hepatic steatosis with no cholelithiasis or acute cholecystitis. The pain still persisted; therefore, the patient had a computed tomographic (CT) scan of the abdomen. Computer tomography imaging of the abdomen and pelvis with intravenous contrast showed acute pancreatitis versus duodenitis (see Figure 1).

**Figure 2:**
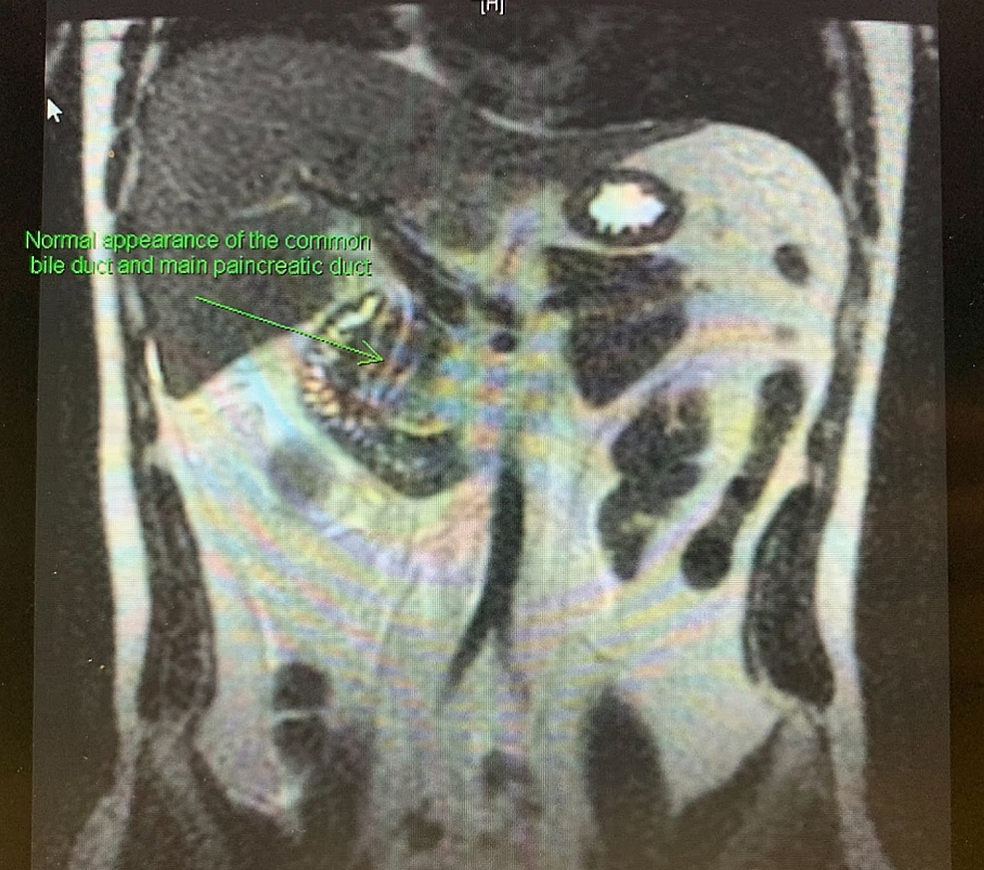
T2-weighted MRCP of the abdomen without fat saturation in a coronal view Magnetic resonance cholangiopancreatography (MRCP) of the abdomen illustrating a normal appearance of the common bile duct and pancreatic duct in our patient.

**Figure 3:**
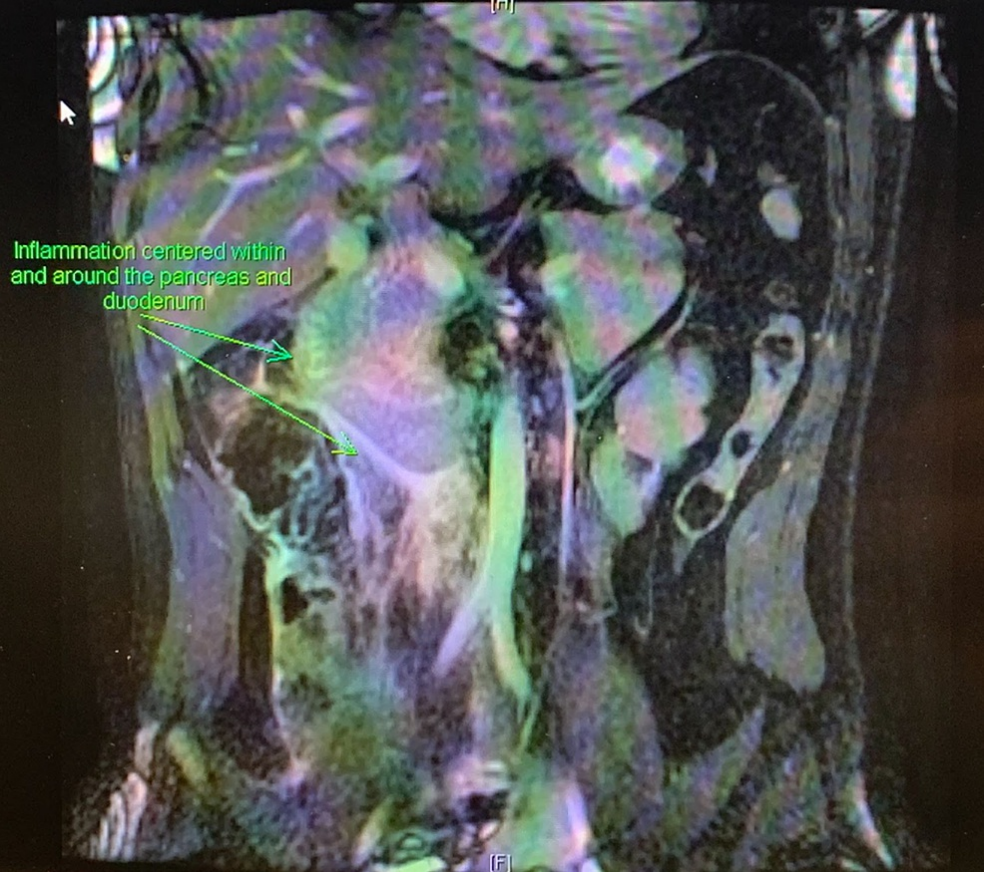
T2-weighted MRCP of the abdomen with fat saturation Coronal view magnetic resonance cholangiopancreatography (MRCP) of the abdomen highlighting inflammation centered within and around the pancreas and duodenum.

Gastroenterology was consulted for pancreatitis of unknown etiology. An MRCP (magnetic resonance cholangiopancreatography) was ordered by the gastroenterologist for further evaluation (see Figure 2 and Figure 3). Follow-up magnetic resonance cholangiopancreatography revealed inflammation within the pancreaticoduodenal groove representing groove pancreatitis with no obstructing stone or ductal dilatation.

In agreement with the gastroenterologist's recommendation, he was treated with supportive care consisting of aggressive isotonic fluid resuscitation, hydromorphone for pain control, lorazepam for anxiety, ondansetron for nausea, docusate sodium and polyethylene glycol for constipation prevention, pantoprazole, and heparin for gastrointestinal and venous thromboembolism prophylaxis, respectively. As for diet, he was on clear liquid as tolerated. His condition gradually improved within six days. The patient felt much better, tolerated oral intake and his lipase began trending down. The patient was pain-free after leaving the hospital and was discharged with instructions to follow up with his primary care physician.

## Discussion

Groove pancreatitis is an extremely rare form of pancreatitis, and there have been seldom descriptions of it in pathology literature [4]. It is increasingly difficult to definitively assess GP regardless of imaging as there is a myriad of differentials such as pancreatic and duodenal cancer, ampullary carcinomas, and gastrointestinal stromal tumors [4]. The presentation and histological characteristics include a thickened pancreatic head and duodenal wall with or without fibrosis. Radiologically, inflammatory changes in the groove between the pancreatic head and duodenum can look similar to a malignancy. However, it is important to consider the whole clinical picture and the patient's symptoms. This patient did not present with weight loss, have a family history of malignancy, or engage in extensive alcohol abuse. Additionally, pancreatic adenocarcinomas are much more likely to invade the retroperitoneum and affect the vasculature, which was not the case with this patient. A more characteristic finding of GP is a thickening of the medial duodenal wall, which is more uncommon with pancreatic adenocarcinoma [4]. Unfortunately, surgical resection in some cases can help differentiate GP and adenocarcinoma, making further treatments and diagnosis profoundly difficult. Patients may undergo a pancreaticoduodenectomy since oftentimes malignancy cannot be excluded. Additionally, from a handful of case reports documented, most patients diagnosed with GP are middle-aged males that are ethanol abusers [5]. This patient presented with a very common gastrointestinal complaint, but with an extremely rare diagnosis. The patient had an elevated lipase, a right upper quadrant ultrasound of hepatic steatosis, and a CT scan of the abdomen acute pancreatitis versus duodenitis. To investigate the cause further, the MRI showed inflammation within the pancreaticoduodenal groove representing GP with no obstructing stone or ductal dilatation, confirming GP. This case is unique in that the patient presented with no risk factors, was young, had no history of alcoholism, thereby making the pathogenesis even more unclear. Although ambiguous, this case may help elucidate and aid the vigilance of a rare diagnosis.

The method of treatment of GP is similar in nature to chronic pancreatitis. General treatment of GP includes intravenous fluids, bowel rest, and opioid analgesia. Additionally, abstaining from alcohol and tobacco should also be considered. His condition improved over time with conservative measures and was eventually discharged to follow up with his family physician and a gastrointestinal doctor.

## Conclusions

Groove Pancreatitis is an uncommon type of chronic pancreatitis associated with duct dysfunction. Diagnosis is often difficult, and many providers are not familiar with the condition, which likely contributes to its low incidence. Appropriately identifying GP will guide the careful decision between performing surgery or proceeding with medical management. While correctly identifying GP can prevent unnecessary surgery, care should be taken to thoroughly rule out pancreatic carcinoma. While medical management is preferred, surgical management is the gold standard in the presence of obstructing symptoms or any suspicion of malignancy. It should therefore be considered as part of the differential for patients presenting with lesions on the pancreatic head.

## References

[1] DeSouza K, Nodit L (2015). Groove pancreatitis: a brief review of a diagnostic challenge. Arch Pathol Lab Med.

[2] Pallisera-Lloveras A, Ramia-Ángel JM, Vicens-Arbona C, Cifuentes-Rodenas A (2015). Groove pancreatitis. Rev Esp Enferm Dig.

[3] Jani B, Rzouq F, Saligram S (2015). Groove pancreatitis: a rare form of chronic pancreatitis. N Am J Med Sci.

[4] Raman SP, Salaria SN, Hruban RH, Fishman EK (2013). Groove pancreatitis: spectrum of imaging findings and radiology-pathology correlation. AJR Am J Roentgenol.

[5] Addeo G, Beccani D, Cozzi D (2019). Groove pancreatitis: a challenging imaging diagnosis. Gland Surg.

